# Extended graph-based models for enhanced similarity retrieval in Cavbase

**DOI:** 10.1186/1758-2946-5-S1-P29

**Published:** 2013-03-22

**Authors:** Timo Krotzky, Thomas Fober, Marco Mernberger, Gerhard Klebe, Eyke Hüllermeier

**Affiliations:** 1 Institute of Pharmaceutical Chemistry, Philipps-Universität, 35032 Marburg/Lahn, Germany; 2 Institute of Mathematics and Computer Science, Philipps-Universität, 35032 Marburg/Lahn, Germany

## 

The problem of estimating the similarity between molecular structures is often tackled by means of graph-based approaches, using graphs for structure representation and measures based on the maximum common subgraph as similarity metrics. In the case of protein binding sites as molecular structures, however, where the graphs can be very large, the computation of these measures may easily become infeasible or at least unacceptably slow.

To this end, Cavbase [1,2] was developed, a database for the automatic detection and storage of putative binding sites on the protein surfaces. Cavbase assigns so-called pseudocenters to the cavity-flanking amino acids, which characterize their physicochemical properties with respect to molecular recognition. On the one side, this representation leads to smaller and more generic representation of a binding site. On the other side, it comes with a loss of information, which is usually compensated by performing further calculations based on additional data. These steps, however, are most often computationally quite demanding, making the whole approach again very slow.

The main drawback of a graph-based model solely based on pseudocenters is the loss of information about the shape of protein surface. In this study, we propose an extended modeling formalism that leads to graphs of the same size, but containing considerably more information. More specifically, additional descriptors of the surface characteristics are extracted from the surface points stored in Cavbase. These properties are included as attributes of the nodes of the graph, which leads to a gain of information and allows for more accurate comparisons between different structures.
